# Zinc Prevents the Development of Diabetic Cardiomyopathy in db/db Mice

**DOI:** 10.3390/ijms18030580

**Published:** 2017-03-07

**Authors:** Shudong Wang, Bowei Wang, Yuehui Wang, Qian Tong, Quan Liu, Jian Sun, Yang Zheng, Lu Cai

**Affiliations:** 1 Cardiovascular Center & Geriatric Medicine, The First Hospital of Jilin University, Changchun 130021, Jilin, China; wangshudong816@163.com (S.W.); tongqian@126.com (Q.T.); liuquan@jlu.edu.cn (Q.L.); sunjian@163.com (J.S.); 2 Pediatric Research Institute, The Department of Pediatrics, University of Louisville, Louisville, KY 40202, USA; wangbw@jlu.edu.cn (B.W.); l0cai001@louisville.edu (L.C.); 3 Gynecology and Obstetrics, The Second Hospital of Jilin University, Changchun 130041, Jilin, China; 4 Wendy Novak Diabetes Care Center, Departments of Pharmacology and Toxicology, University of Louisville, Louisville, KY 40202, USA

**Keywords:** diabetic cardiomyopathy, zinc supplement, nuclear factor-erythroid 2-related factor 2, inflammation, oxidative stress

## Abstract

Diabetic cardiomyopathy (DCM) is highly prevalent in type 2 diabetes (T2DM) patients. Zinc is an important essential trace metal, whose deficiency is associated with various chronic ailments, including vascular diseases. We assessed T2DM B6.BKS(D)-Leprdb/J (db/db) mice fed for six months on a normal diet containing three zinc levels (deficient, adequate, and supplemented), to explore the role of zinc in DCM development and progression. Cardiac function, reflected by ejection fraction, was significantly decreased, along with increased left ventricle mass and heart weight to tibial length ratio, in db/db mice. As a molecular cardiac hypertrophy marker, atrial natriuretic peptide levels were also significantly increased. Cardiac dysfunction and hypertrophy were accompanied by significantly increased fibrotic (elevated collagen accumulation as well as transforming growth factor β and connective tissue growth factor levels) and inflammatory (enhanced expression of tumor necrosis factor alpha, interleukin-1β, caspase recruitment domain family member 9, and B-cell lymphoma/leukemia 10, and activated p38 mitogen-activated protein kinase) responses in the heart. All these diabetic effects were exacerbated by zinc deficiency, and not affected by zinc supplementation, respectively. Mechanistically, oxidative stress and damage, mirrored by the accumulation of 3-nitrotyrosine and 4-hydroxy-2-nonenal, was significantly increased along with significantly decreased expression of Nrf2 and its downstream antioxidants (NQO-1 and catalase). This was also exacerbated by zinc deficiency in the db/db mouse heart. These results suggested that zinc deficiency promotes the development and progression of DCM in T2DM db/db mice. The exacerbated effects by zinc deficiency on the heart of db/db mice may be related to further suppression of Nrf2 expression and function.

## 1. Introduction

Diabetic cardiomyopathy (DCM) is highly prevalent in asymptomatic type 2 diabetes (T2DM) patients. Diabetes is associated with an increased risk of developing heart failure; indeed, 75% of patients with unexplained idiopathic dilated cardiomyopathy were found to be diabetic [1]. Metabolic abnormalities, including hyperglycemia, hyperinsulinemia, and hyperlipidemia, can lead to cellular alterations such as myocardial fibrosis and/or myocardial hypertrophy, and, eventually, DCM. Several studies, mainly echocardiographic and population-based studies, have documented a uniform association of DCM with cardiac hypertrophy and myocardial stiffness, independently of hypertension. The mechanisms underlying the pathophysiology of this disease are not completely elucidated.

It is well known that oxidative stress and inflammation are involved in the pathogenesis of cardiovascular diseases [2]. Extensive research in the past two decades has revealed the mechanism by which continued oxidative stress causes chronic inflammation, which in turn mediates most chronic diseases, including cancer and diabetes, as well as cardiovascular, neurological, and pulmonary ailments. Oxidative stress can activate a variety of transcription factors, including nuclear factor-erythroid 2-related factor 2 (Nrf2) [2,3]. Nrf2 plays an important role in preventing oxidative damage, which contributes to cardiac inflammation. The role of Nrf2 in preventing diabetes-induced oxidative stress is also established. First, Nrf2 is quickly up-regulated in tissues or cells in response to hyperglycemia or high glucose levels [4,5]. Second, diabetes-induced cardiac and renal damage is more severe in *Nrf2* gene knockout mice than in wild-type (WT) counterparts [4,6,7]. Third, Nrf2 activation by sulforaphane in vitro and in vivo or MG132 in vivo suppresses high glucose-induced ROS production and metabolic dysfunction in human microvascular endothelial cells [8] and attenuates diabetic proteinuria in streptozotocin (STZ)-induced diabetic rats [9,10]. Sulforaphane treatment that activates renal Nrf2 function induces renal protection only in WT mice, not in animals with *Nrf2* gene deletion [10].

Zinc (Zn) is an important essential trace metal, a deficiency of which promotes the development of cardiovascular diseases in humans [11]. Studies assessing animal models have also found an association between Zn deficiency and various vascular diseases [11,12]. Increasing evidence from human and animal studies shows an effect of Zn on diabetic complications, seemingly through Nrf2 induction by Zn [13,14].

We previously demonstrated that a high-fat diet (HFD) induces a time-dependent obesity as well as obesity-related cardiac hypertrophy, accompanied by increased cardiac inflammation and p38 MAPK activation. Zn supplementation alleviates, while its deficiency heightens cardiac hypertrophy in HFD-induced obese mice, by suppressing p38 MAPK-dependent cardiac inflammatory and hypertrophic pathways [15]. We also demonstrated that Zn protects against T1DM-induced damage to the kidney, liver, and testis, mainly by increasing insulin-like function and reducing oxidative stress and inflammation [16,17]. However, whether Zn has similar effects on the heart in T2DM remains unclear.

In the present study, therefore, we tested the following hypotheses: (1) Zn deficiency may accelerate the development and progression of DCM in T2DM; (2) mechanistically, Zn deficiency may impair the expression and function of Nrf2, leading to an exacerbation of diabetes-induced pathogenic process in cardiomyopathy. Because the effects of Zn on leptin expression have been extensively reported [18,19], we asked whether Zn deficiency exacerbates and its supplementation attenuates HFD/obesity-induced cardiac alterations by reducing or increasing leptin levels. To this end, T2DM B6.BKS(D)-Leprdb/J (db/db) mice with leptin receptor deletion were used to rule out the potential effect of Zn deficiency and supplementation on obesity-induced cardiac pathogenesis via systemic leptin signaling. In addition, whether the exacerbated or inhibitory effects of Zn deficiency and supplement on the heart are related to HFD components remains unclear. Consequently, we did not include HFD since db/db mice still develop obesity on a normal diet; this approach would eliminate the potential direct effects of HFD-contained components on the heart.

## 2. Results

### 2.1. General Features of db/db Mice after Treatment with Different Zn Amounts

**Figure 1:**
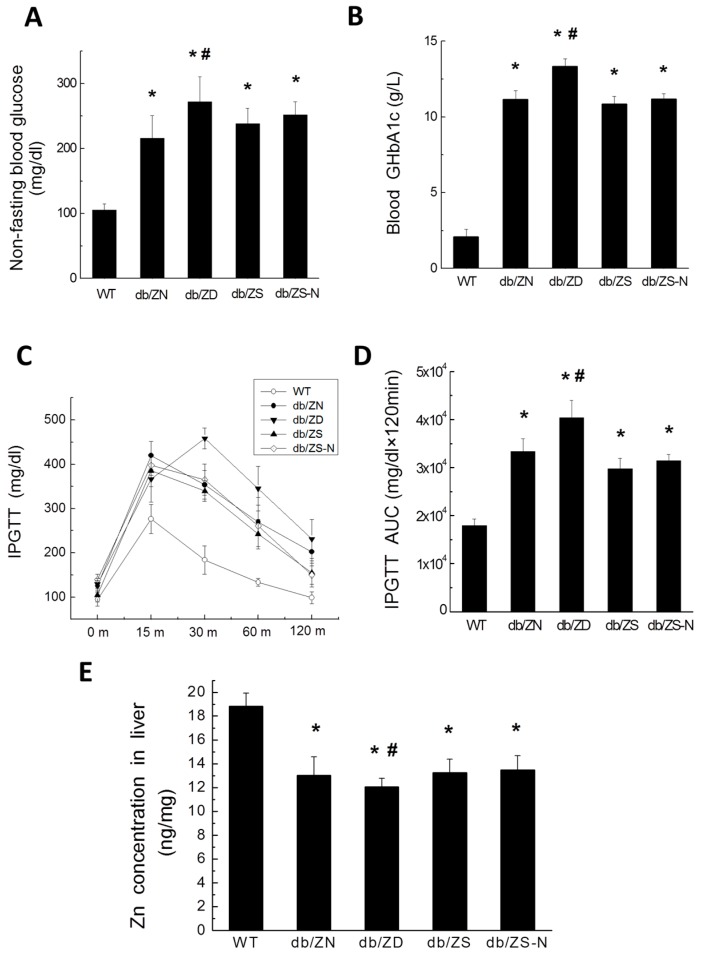
Systemic effects of different Zn amounts in db/db mice. Twenty-one B6.BKS(D)-Leprdb/J mice were randomly divided into four groups and fed normal chow (10% Cal from fat) with different amounts of Zn (ZD, ZN, ZS and ZS-N, respectively); seven C57BL/6J mice were fed ZN as the control group (WT). All mice were fed from the age of 17 weeks, and sacrificed six months later (as 6M). Non-fasting glucose levels (**A**) and blood GHbA1c (g/L) (**B**) were evaluated. IPGTT (**C**) at six months and related area under curve (AUC) values (**D**) were assessed. Zn levels in the liver were measured in WT and diabetic mouse groups (**E**). Data were presented as mean ± SD (*n* = 7 for WT control and db/db-ZD (db/ZD) groups; *n* = 6 for db/db-ZN (db/ZN) group; *n* = 4 for db/db-ZS (db/ZS) and db/db-ZS-N (db/ZS-N) groups). Differences were assessed by ANOVA with Tukey–Kramer post hoc analysis. *, *p* < 0.05 vs. WT group; #, *p* < 0.05 vs. db/ZN group.

Mice were fed normal diet with different amounts of Zn, including Zn deficient (ZD), Zn adequate (ZN), Zn supplemented (ZS), and ZS for the first three months and switched to ZN (ZS-N) groups, starting at the age of 17 weeks. Non-fasting blood glucose levels (Figure 1A) and blood GHbA1c (Figure 1B) were increased in db/db mice compared with WT animals. Insulin resistance was assessed by intraperitoneal glucose tolerance test (IPGTT) with injection of 2 g/kg body weight, and blood glucose was assessed at the six month time point (Figure 1C), followed by AUC determination (Figure 1D). db/db mice showed significantly increased IPGTT, as reflected by elevated AUCs at six months (Figure 1C,D). Interestingly, ZD worsened the db/db mouse condition, further increasing non-fasting blood glucose levels, blood GHbA1c, and IPGTT, while ZS and ZS-N showed no significant changes compared with the ZN group. Calorie intake was not significantly changed based on daily food consumption.

### 2.2. Zn Levels in Cardiac and Liver Tissues

Because cardiac tissue and serum specimens were limited, we measured hepatic Zn levels. As shown in Figure 1E, hepatic Zn levels in db/db mice were decreased significantly compared with the amounts of WT mice. Compared with the ZN group, the ZD group showed further decreased levels of hepatic Zn, while no significant change was found in the ZS and ZS-N groups.

### 2.3. Cardiac Hypertrophy and Function

**Figure 2:**
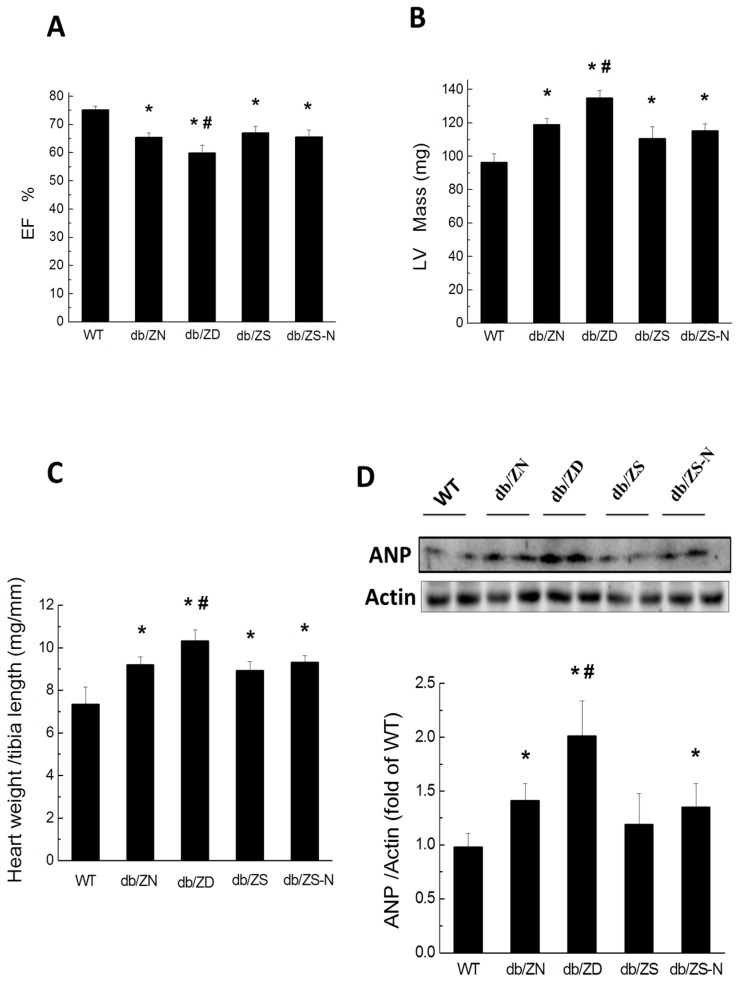
Zn deficiency exacerbates diabetic-induced heart hypertrophy and function. Animals were treated as described in Figure 1. EF% (**A**) and corrected Left ventricular (LV) mass (mg/g) (**B**) were examined by echocardiography. Heart-weight to tibial length ratio (**C**) and atrial natriuretic peptide (ANP) protein levels obtained by Western Blot (**D**) were assessed as indicators of heart hypertrophy and function. Data were presented as mean ± SD (*n* = 4–7, details in Figure 1). *, *p* < 0.05 vs. WT group; #, *p* < 0.05 vs. db/ZN group.

Echocardiographic analysis showed that EF% (Figure 2A) in db/db mice decreased significantly, with markedly increased LV mass (Figure 2B) and heart weight to tibial length ratio (Figure 2C). ANP protein expression levels were significantly higher in db/db mice than in WT animals (Figure 2D). All these parameters were significantly worsened by ZD, compared with the ZN group, but not significantly changed in the ZS and ZS-N groups.

### 2.4. Zn Prevents Cardiac Fibrosis in db/db Mice

**Figure 3:**
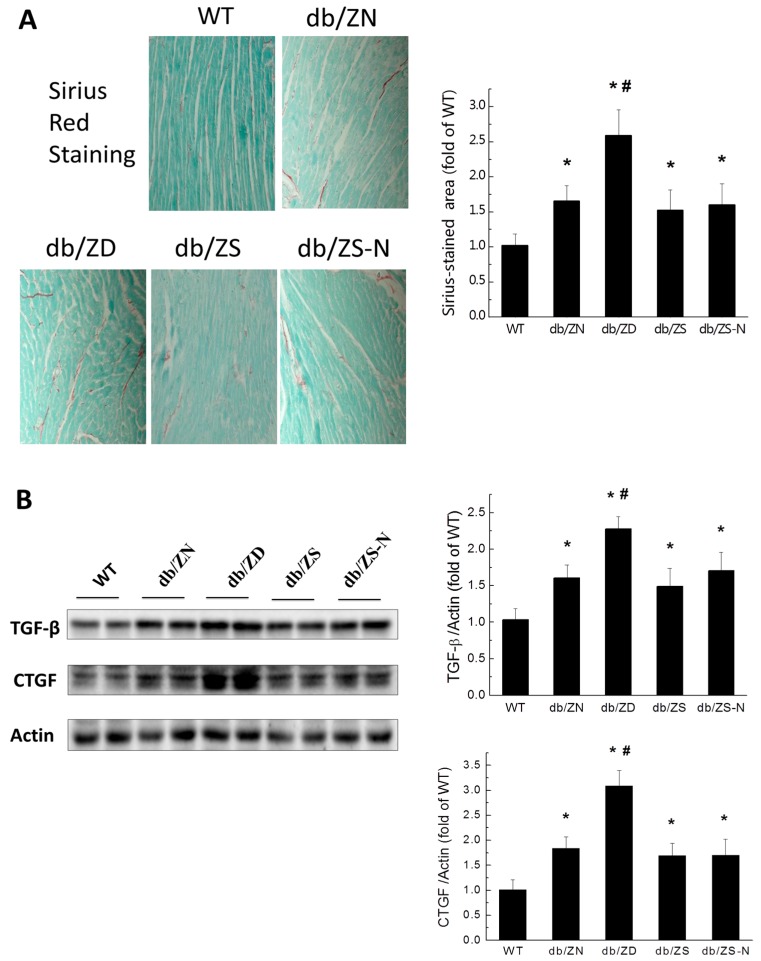
Zn deficiency exacerbates diabetes-induced cardiac fibrosis. Sirius Red Staining (**A**) was used to assess fibrosis in the heart, Scale bar = 25 µM. Western blot was used to evaluate fibrosis related factors (**B**), including transforming growth factor β (TGF-β) and connective tissue growth factor (CTGF). Data were presented as mean ± SD (*n* = 4–7, details in Figure 1). *, *p* < 0.05 vs. WT group; #, *p* < 0.05 vs. db/ZN group.

Sirius Red Staining followed by semi-quantitative analysis (Figure 3A) indicated increased fibrosis in all four db/db groups compared with WT controls. Among the db/db mice, the db/db-ZD group had worse outcome compared with db/db-ZN animals, while the ZS and ZS-N groups showed no significant change in terms of cardiac fibrosis severity compared to the ZN group. Western blot demonstrated that TGF-β and CTGF amounts in the diabetic groups were significantly higher compared with WT group levels (Figure 3B). Meanwhile, db/db-ZD animals had significantly higher expression levels of TGF-β and CTGF compared with the db/db-ZN group. However, the ZS, ZN and ZS-N groups showed similar TGF-β and CTGF levels.

### 2.5. Zn Prevents Cardiac Inflammation in db/db Mice

**Figure 4:**
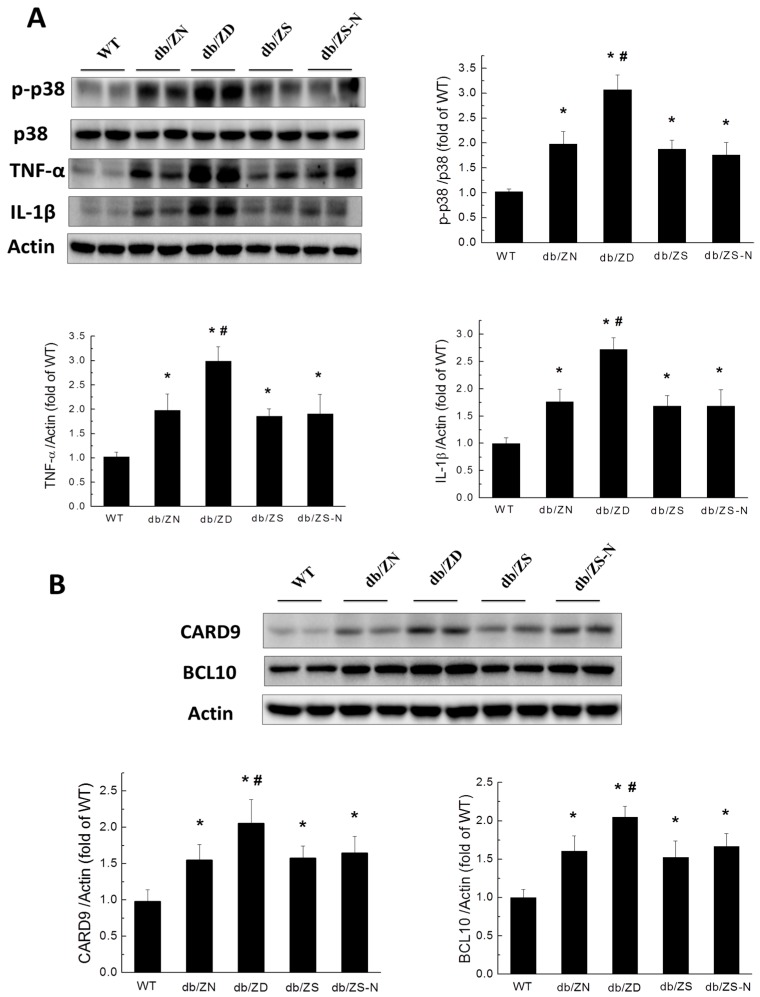
Zn deficiency exacerbates diabetes-induced cardiac inflammation. Western blot was used to assess fibrosis-related factors such as p-P38/p38, TNF-α, IL-1β (**A**), CARD9, and BCL10 (**B**). Data were presented as mean ± SD (*n* = 4–7, details in Figure 1); differences were assessed by ANOVA with Tukey–Kramer post hoc analysis. *, *p* < 0.05 vs. WT group; #, *p* < 0.05 vs. db/ZN group.

Given that hyperglycemia induces chronic inflammation as a causative factor of cardiac remodeling, the expression levels of inflammatory markers, including p-P38, p38, TNF-α, IL-1β (Figure 4A), CARD9, and BCL10 (Figure 4B), were examined by Western blot. Interestingly, diabetic mice showed significantly increased expression levels of these proteins in cardiomyopathy at the 6M time-point; these changes were even more notable in the ZD group. However, Zn supplementation (ZS and ZS-N) alleviated these effects, and the amounts of the above inflammatory factors were similar to ZN group values.

### 2.6. Zn Attenuation of Cardiac Oxidative Stress Is Probably Associated with Nrf2 Activation to Upregulate Downstream Antioxidants

**Figure 5:**
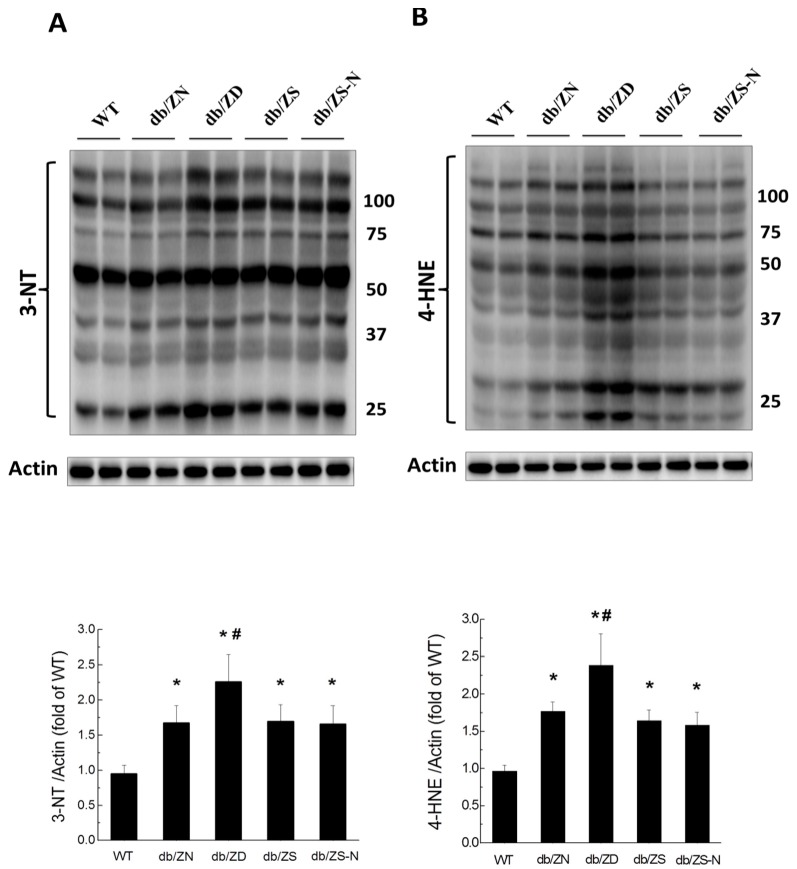
Zn exacerbates diabetes-induced cardiac oxidative damage. Oxidative damage was examined by Western blot for 3-NT (**A**) and 4-HNE (**B**) accumulation in the myocardium, followed by quantitative analysis. 3-NT includes all nitrated tyrosine containing proteins (**A**). Similarly, 4-HNE includes all proteins containing 4-hydroxy-2-nonenal (a peroxide lipid) (**B**). All bands were quantified. Data were presented as mean ± SD (*n* = 4–7, details in Figure 1). *, *p* < 0.05 vs. WT group; #, *p* < 0.05 vs. db/ZN group.

Considering that inflammation is often accompanied by oxidative stress, such damage was evaluated by Western blot for 3-NT (Figure 5A) and 4-HNE (Figure 5B); significantly increased amounts of these proteins were found in diabetic mice at the 6M time-point. ZD exacerbated cardiac oxidative stress in diabetic mice, while the ZS and ZS-N groups showed 3-NT and 4-HNE levels similar to ZN group values.

**Figure 6:**
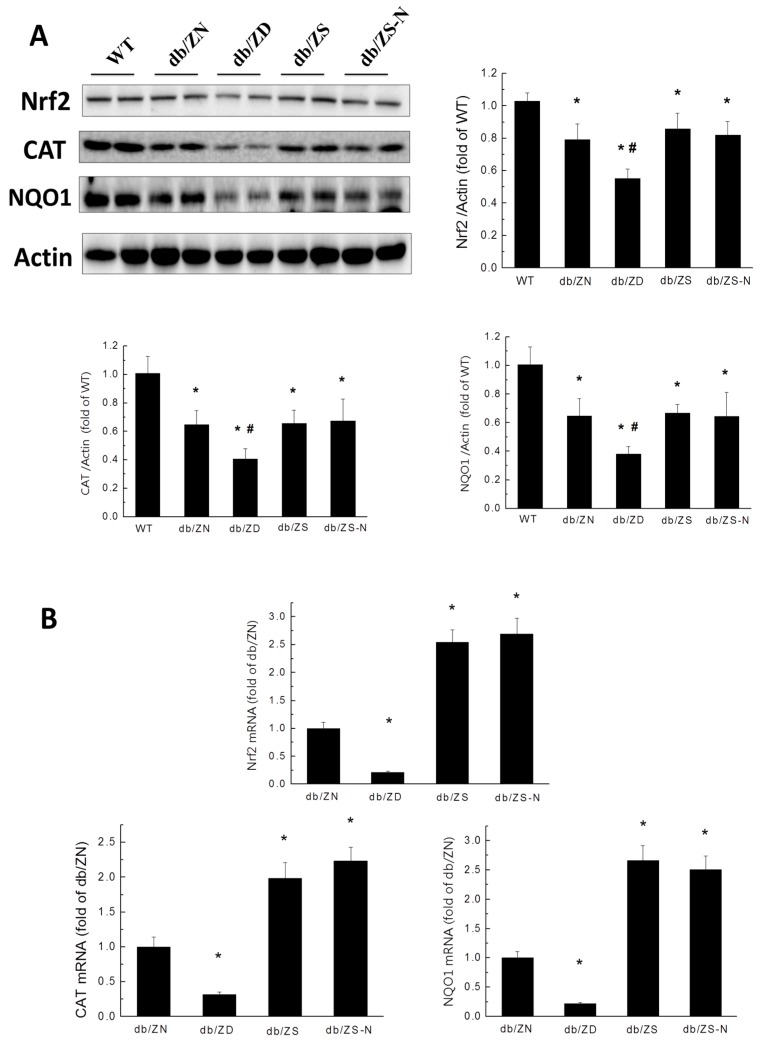
Effects of Zn deficiency and supplementation on T2DM-induced downregulation of Nrf2 and downstream genes in db/db mice. The expression levels of Nrf2 protein and downstream factors, such as catalase and NAD(P)H dehydrogenase 1( NQO-1), were examined by Western blot (**A**) and RT-PCR (**B**). Data were presented as mean ± SD (*n* = 4–7, details in Figure 1). *, *p* < 0.05 vs. WT group; #, *p* < 0.05 vs. db/ZN group.

To explore the mechanism behind the protective effects of Zn toward diabetic-induced pathological changes in the mouse heart, nuclear transcription factor (Nrf2) expression was analyzed at the protein and gene levels, respectively, by Western blot and RT-PCR. Western blot showed that db/db mice displayed significantly decreased amounts of Nrf2 and the downstream catalase (CAT) and NQO1 proteins in cardiac tissues. Zn deficiency aggravated these changes, while the ZS and ZS-N groups showed similar protein levels of Nrf2, CAT, and NQO1 to the ZN group (Figure 6A). At the transcriptional level, mRNA amounts of *Nrf2*, catalase and *NQO-1* were significantly decreased in diabetic mice of the ZD group, while the ZS and ZS-N groups showed increased mRNA levels of these genes (Figure 6B).

## 3. Discussion

Both diabetes and Zn deficiency are global health problems [1,11,20]. Diabetic patients often suffer from Zn deficiency at the late disease stage, particularly those whose glucose is poorly controlled [21,22,23,24]. Zn supplementation has beneficial effects on glucose and lipid control [25]. In the present study, an animal model of T2DM with leptin receptor defect was used to demonstrate that Zn deficiency significantly exacerbates obesity-induced cardiac oxidative damage, inflammation, and fibrosis in diabetes, effects associated with significantly decreased expression of Nrf2 and the downstream antioxidants NQO-1 and CAT. These changes were exacerbated by Zn deficiency, but not affected by Zn supplementation in db/db mice. As an adaptive mechanism, Nrf2 is quickly upregulated in cells and tissues in response to various oxidative stresses, but downregulated at late stage after exposure to chronic oxidative stress [17,26,27,28]. In the current study, cardiac Nrf2 expression levels were decreased in diabetic mice after six months on HFD, probably because the diabetes duration was long enough. Moreover, diabetic mice with Zn deficiency showed further downregulation of Nrf2 and the downstream effectors NQO-1 and catalase. More importantly, Zn deficiency-exacerbated Nrf2 downregulation resulted in severely increased oxidative damage, cardiac inflammation, and fibrosis. Meanwhile, an important finding was that Zn deficiency aggravated diabetes-induced pathogenic changes, in association with further Nrf2 downregulation in the T2DM model.

Multiple pathways have been linked to hyperglycemia-induced oxidative stress, inflammation, fibrosis, and cardiovascular diseases. T2DM has been experimentally characterized by myocardial inflammation, including increased TNF-α expression, oxidative stress, and fibrosis [29]. Phosphor-p38 MAPK upregulation was reported to contribute to cardiac hypertrophy in obese [15] and T2DM [30,31] animals. BCL10 reportedly binds CARD9 to activate NF-κB [32]. BCL10 deletion resulted in reduced cardiac remolding induced by angiotensin II [33]; similarly, CARD9 deletion also ameliorated obese-induced cardiac dysfunction [31].

However, other studies demonstrated that CARD9 mediates the activation of p38 MAPK, which is pivotal in multiple immune responses and inflammation activation [34,35,36]. CARD9 signaling allows Toll like receptor (nucleotide-binding oligomerization domain) pathways to induce p38 MAPK activation [34,37]. Previous studies reported that CARD9 knockout attenuates cardiac dysfunction by abrogating increased p38 MAPK phosphorylation in obese mice, as well as cardiac inflammation and fibrosis after angiotensin II treatment [31,38]. The present study demonstrated that Zn deficiency exacerbated BCL10 and CARD9 expression changes in db/db mice, although there were no significant differences among the ZN, ZS, and ZS-N groups.

Our team and others have reported that overexpression of metallothionein, catalase, and manganese superoxide dismutase in the heart reverses diabetic cardiomyopathy in animal models of both T1DM and T2DM [39,40,41,42]. Thus, strategies that either reduce ROS or augment myocardial antioxidant defense mechanisms might have therapeutic efficacy in improving myocardial function in diabetes. As shown above, db/db mice displayed significantly decreased levels of Nrf2 and downstream antioxidants, including catalase, compared with WT control mice. Among the db/db mice, the expression levels of Nrf2 and downstream antioxidants were lowest in the ZD group, which may be the main reason why Zn deficiency exacerbates obesity/T2DM-induced cardiac remodeling and dysfunction. Indeed, low antioxidant capacity may lead to high oxidative stress that induces BCL10/CARD9-mediated p38 MAPK activation, resulting in cardiac inflammation and remodeling, and even dysfunction. In addition, since the ZD group also showed worst outcomes of blood glucose and GHbA1c levels as well as insulin resistance, the worsening effects of ZD on these systemic alterations may also be in part responsible for the aggravated development of DCM.

It should be mentioned that, in the present study, Zn supplementation to db/db mice did not exert any beneficial effects on T2DM-induced-cardiac pathogenesis compared to the ZN group. In contrast, we recently demonstrated that HFD induces cardiac hypertrophy along with inflammation, which were exacerbated and attenuated by Zn deficiency and supplementation, respectively, compared with ZN groups, in HFD-induced obese WT mice [15]. The discrepancy between the two studies suggests that Zn supplementation-mediated cardiac protection from obesity and/or T2DM is likely dependent on leptin-mediated signaling, as shown by the impact of Zn on leptin levels [18,19]; this will be further explored in future studies.

## 4. Materials and Methods

### 4.1. Animals

B6.BKS(D)-Leprdb/J (db/db) and C57BL/6J mice were obtained from the Jackson Laboratory (Bar Harbor, ME, USA), and housed in the University of Louisville Research Resources Center at 22 °C under a 12:12 h light/dark cycle, with tap water and rodent diet ad libitum. All experimental procedures were approved by the Institutional Animal Care and Use Committee of the University of Louisville (3 April 2015), and carried out in accordance with the Guide for the Care and Use of Laboratory Animals, Eighth Edition (Library of Congress Control Number: 2010940400, revised 2011). Twenty-one B6.BKS (D)-Leprdb/J mice were randomly divided into four groups. The first 17 mice were fed a normal diet (10% calories from fat) with different amounts of Zn, including 10 mg (deficient, ZD; *n* = 7), 30 mg (adequate, ZN; *n* = 6), and 90 mg (supplemented, ZS; *n* = 4) per 4057 Kcal, respectively, for six months. The fourth group of four db/db mice were fed ZS for the first three months and switched to ZN for the subsequent three months, the same as the db/db/ZS-N group. Meanwhile, seven C57BL/6J mice were fed a ZN diet for six months as a control group. After six months, all mice were sacrificed at 6M.

### 4.2. Intraperitoneal Glucose Tolerance Test (IPGTT)

Insulin resistance was assessed by IPGTT [43]. Briefly, mice were fasted for 6 h (8:00 a.m.–2:00 p.m.) and injected intraperitoneally with d-(+)-glucose (Sigma-Aldrich, St. Louis, MO, USA) at a dose of 2 g/kg body weight. Blood glucose levels at 0, 15, 30, 60, and 120 min after glucose injection were measured using a FreeStyle Lite glucometer (Abbott Diabetes Care, Alameda, CA, USA). Glucose tolerance test area under the curve (AUC) was assessed by the trapezoid rule with the Origin 8.6 software (OriginLab Co., Northampton, MA, USA).

### 4.3. Echocardiography for EF% and LV Mass Assessment

Transthoracic echocardiography was performed as described previously [44]. Briefly, a high-resolution imaging system for small animals (Vevo 770, Visual Sonics, Toronto, Ontario, Canada) equipped with a high-frequency ultrasound probe (RMV-707B) was used. Left ventricular (LV) dimensions, end-diastolic inter-ventricular septum thickness (IVS; d), end-diastolic left ventricular posterior wall thickness (LVPW; d), LV fractional shortening (FS), LV mass, and LV ejection fraction (EF) were measured from LV M-Mode images.

### 4.4. Zn Concentration Assessment in the Liver Tissue

Each liver sample (30 mg wet-weight) was digested with 1 mL 70% concentrated nitric acid, in an 85 °C water bath for 3 h. After digestion, each sample was diluted 35 times. The digested samples were then filtered using a PTFE 0.2 mm filter. Liver Zn levels were assessed by Atomic Absorption Spectroscopy (AAS) on an iCE-3000 AAS instrument from Thermo Fisher Scientific (Waltham, MA, USA). Zn levels were calculated based on a standard curve and presented as ng/mg wet tissue.

### 4.5. Sirius Red Staining

Heart fibrosis was assessed by Sirius red staining for collagen, with a mixture of 0.1% Sirius red F3BA and 0.25% Fast Green FCF. Collagen amounts in the myocardium were evaluated using Pro Plus 6.0 software (Media Cybernetics Inc., Bethesda, MD, USA).

### 4.6. Quantitative Real-Time PCR

Total RNA was extracted from heart tissues with TRIzol-reagent (RNA STAT60 Tel-Test; Austin, Texas, USA). RNA amounts and purity were determined on a Nanodrop ND-1000 spectrophotometer (Thermo Scientific, Wilmington, DE, USA). Random-primed strand complimentary DNA (cDNA) was synthesized from total RNA using GoScript Reverse Transcription System (Promega, Madison, WI, USA) following the manufacturer’s instructions. Primers for NQO-1 (Mm01253561_m1), catalase (Mm00437992_m1), Nfe2/2 (Mm00477784_m1), and glyceraldehyde-3-phosphate dehydrogenase (GAPDH, Mm99999915_g1) were purchased from Thermo Fisher Scientific. Quantitative RT-PCR (qRT-PCR) was performed in duplicate in a 20 µL reaction system comprising 10 µL of TaqMan Universal PCR Master Mix, 1 µL of each primer, and 3 µL of cDNA, on an ABI 7500 RT-PCR system (Applied Biosystems, Foster City, CA, USA). *C*_t_ values were obtained, and relative gene expression was assessed by the 2^−ΔΔ*C*t^ method, with GAPDH used for normalization.

### 4.7. Western Blot

Heart tissues were homogenized in RIPA lysis buffer (sc-24948A, Santa Cruz Biotech, CA, USA). After centrifugation at 12,000 *g* and 4 °C for 15 min, the supernatants were collected and protein levels determined using the Bradford protein assay (Bio-Rad, Hercules, CA, USA). Tissue lysates were diluted with loading buffer and heated to 95 °C for 5 min. Then, equal amounts of total protein (40 µg) were separated by 10% SDSPAGE at 120 V and transferred onto 0.2 µm nitrocellulose membranes (162-0112, Bio-Rad) for 1.5 h at 4 °C. Nonspecific proteins were blocked with 5% nonfat dried milk and 0.5% BSA in TBS-T (20 mM Tris–HCl [pH 7.6], 150 mM NaCl, and 0.1% Tween 20) for 1 h at room temperature with agitation. Subsequently, the membranes were incubated with anti-tumor necrosis factor α (TNF-α, Abcam, Cambridge, USA, ab6671, 1:1000), anti-connective tissue growth factor (CTGF1, Santa Cruz Biotech., sc-14939, 1:1000), anti-phospho-p38 (Thr180/Tyr1821, Cell Signaling, Danvers, MA, 1:1000) , anti-p38 (Cell Signaling, 1:1000), anti-atrial natriuretic peptide (ANP, Santa Cruz Biotech, 1:1000), anti-interleukin 1β (IL-1β, Santa Cruz Biotechnology, sc-7884, 1:1000), anti-caspase recruitment domain family member 9 (CARD9, Cell Signaling, #12283, 1:1000), anti-B-cell lymphoma/leukemia 10 (BCL10, Santa Cruz Biotechnology, sc-5611, 1:1000), anti-3-nitrotyrosine (3-NT, Millipore, 1:3000), anti-4-hydroxy-2-nonenal (4-HNE, Alpha Diagnostic International, San Antonio, TX, USA, 1:3000), and anti-β-actin (Santa Cruz Biotech., sc1616, 1:3000) primary antibodies overnight at 4 °C. After three washes with TBS-T, the membranes were incubated with HRP-conjugated secondary antibodies (Santa Cruz Biotech) for 1 h at room temperature. Immunoreactive bands were visualized using an enhanced chemiluminescence (ECL) kit (Bio-Rad), and revealed on a ChemiDoc Touch Imaging System (Bio-Rad). Protein expression levels were normalized to β-actin as an internal control. For Western blot, aortic tissues were homogenized in a RIPA lysis buffer (sc-24948A, Santa Cruz Biotech.).

### 4.8. Statistical Analysis

Data are mean ± standard deviation (SD) with 4–7 animals per group. Group comparisons were performed by one-way analysis of variance (ANOVA), followed by pairwise repetitive comparisons with Tukey test, using the Origin 8.0 Lab data analysis and graphing software. *p* < 0.05 was considered statistically significant.

## 5. Conclusions

In conclusion, this is the first study to our knowledge providing evidence regarding the relationship between DCM and long-term Zn treatment in T2DM animal models. We confirmed that Zn deficiency contributes to the pathological progression of cardiac disorders induced by T2DM, and revealed the partial protective effect of long-term Zn supplementation on DCM. These findings confirm the significant role of Zn in preventing diabetic related cardiac disorders in T2DM mouse models. Optimal Zn supplementation dose for T2DM mice could be identified to prevent DCM. Overall, Zn supplements might be highly potent in the prevention and/or treatment of T2DM related DCM.
